# Trends in Small-Cell Lung Cancer Survival in 1993–2006 Based on Population-Based Cancer Registry Data in Japan

**DOI:** 10.2188/jea.JE20180112

**Published:** 2019-09-05

**Authors:** Isao Oze, Hidemi Ito, Yoshikazu Nishino, Masakazu Hattori, Tomio Nakayama, Isao Miyashiro, Keitaro Matsuo, Yuri Ito

**Affiliations:** 1 Division of Cancer Epidemiology and Prevention, Department of Preventive Medicine, Aichi Cancer Center Research Institute, Nagoya, Japan; 2 Division of Cancer Information and Control, Department of Preventive Medicine, Aichi Cancer Center Research Institute, Nagoya, Japan; 3 Department of Epidemiology, Nagoya University Graduate School of Medicine, Nagoya, Japan; 4 Department of Epidemiology and Public Health, Kanazawa Medical University, Ishikawa, Japan; 5 Department of Cancer Therapy Center, Fukui Prefectural Hospital, Fukui, Japan; 6 Division of Screening Assessment and Management, Screening Research Group, Center for Public Health Sciences, National Cancer Center, Tokyo, Japan; 7 Cancer Control Center, Osaka International Cancer Institute, Osaka, Japan; 8 Department of Medical Statistics, Research & Development Center, Osaka Medical College, Takatsuki, Osaka, Japan

**Keywords:** cancer registry, population-based, small cell lung cancer, survival

## Abstract

**Background:**

Lung cancers are classified into small-cell lung cancer (SCLC) and non-small-cell lung cancer due to their different treatment and prognosis. Although many studies have reported the specific survival of SCLC patients treated at cancer hospitals, survival from population-based data has rarely been reported.

**Methods:**

We analyzed survival of SCLC cases diagnosed from 1993 through 2006 from a population-based cancer registry of six prefectures. To assess trends in SCLC survival, we defined three periods that mirrored developments in SCLC treatment: period 1, 1993–1998; period 2, 1999–2001; and period 3, 2002–2006. Assessments were based on relative survival (RS), excess hazard, and conditional survival.

**Results:**

A total of 10,911 SCLC patients were analyzed. Five-year RS among limited disease SCLC (LD-SCLC) in periods 1 to 3 was 16.8%, 21.1%, and 21.4%, respectively. Five-year RS among extensive disease SCLC (ED-SCLC) in periods 1 to 3 was 2.3%, 2.8%, and 2.7%, respectively. Improvement in 5-year RS in periods 2 and 3 compared with period 1 was significant among both LD- and ED-SCLC patients (all *P* < 0.001). Conditional 5-year RS of LD-SCLC increased from 21% at year 0 to 73% at year 5, while that of ED-SCLC was 3% at year 0 and 53% at year 5.

**Conclusions:**

The prognosis of SCLC patients improved from 1999–2001 but plateaued in 2002–2006, after which no further significant improvement was seen. Continuous survey based on population-based data is helpful in monitoring the impact of developments in treatment.

## INTRODUCTION

Lung cancers are classified into two broad classes, small-cell lung cancer (SCLC) and non-small-cell lung cancer (NSCLC).^1^^,^^2^ These cancers differ biologically and, accordingly, also differ in their therapy and prognosis.^3^ National rates of survival of total lung cancer patients have been reported for countries all over the world,^4^ and while reporting of histologic subtype-specific survival of lung cancer patients treated in cancer hospitals is also common,^5^^,^^6^ reporting of this survival from population-based data is rare. With regard to SCLC status, however, this may be problematic for three reasons: cancer patients treated in cancer hospitals have a relatively better health status than those treated at general hospitals; survival reports from cancer hospitals are often restricted to patients who undergo surgery; and overall lung cancer survival from population-based data mainly reflects the survival of patients with NSCLC, given that NSCLC accounts for more than 80% of lung cancer cases.^7^ For these reasons, overall lung cancer survival data might not be applicable to patients with SCLC.

Prognosis of cancer patients is modified by disease stage and treatment.^8^ Treatment plans in patients with SCLC are commonly determined using a two-stage system originally introduced by the Veterans’ Affairs Lung Study Group, together with the TNM staging system.^9^^,^^10^ SCLC patients are classified into two stages, limited disease (LD) or extensive disease (ED), which are utilized for treatment selection. Tumor confined to the ipsilateral hemithorax and regional nodes is defined as LD, and tumor beyond the boundaries of LD is defined as ED. In general, patients with LD-SCLC are treated using multimodal treatment, while those with ED-SCLC receive systemic therapy.^11^

SCLC treatment has changed over time. Around 1999, several clinical studies supported the efficacy of concurrent chemoradiotherapy and hyperfractionated radiotherapy for LD-SCLC.^12^^,^^13^ The efficacy of new combination chemotherapy with cisplatin and irinotecan for Japanese patients with ED-SCLC was established in 2002.^14^ In addition, new drugs for ED-SCLC, amrubicin and topotecan, were approved in Japan in 2002 and 2003, respectively.^15^^–^^17^ Although these developments in SCLC treatment might have improved prognosis, scarce evidence for their impact is available based on population-based data.

Here, to determine specific survival of SCLC with consideration to disease stage and developments in treatment, we estimated recent trends in 10-year survival of patients with SCLC based on population-based data in Japan.

## MATERIAL AND METHODS

### Data source

This study was conducted using the framework of the Japanese Cancer Survival Information for Society (J-CANSIS) study. Details of the J-CANSIS study are provided elsewhere.^18^ In brief, the J-CANSIS study aimed to analyze recent trends in cancer survival and report long-term survival based on population-based cancer registry data of six prefectures (Yamagata, Miyagi, Fukui, Niigata, Osaka, and Nagasaki) in Japan. These six registries provided a total of 98,475 lung cancer cases diagnosed between 1993 and 2006. The population covered in our study represents 13.4% of the total Japanese population and includes both urban and rural areas. These prefectural cancer registries have high data quality (% of death certificate only = 3.1–24.6%) and have long been used to estimate national statistics for cancer survival in Japan.^19^ Morphologies of lung cancer were recorded using the morphology codes of the International Classification of Diseases for Oncology, Third Edition (ICD-O-3).^20^ Data from cancer patients followed for 5 years or more were used. Patients were linked to the prefecture death certificate database to confirm their vital status. The Yamagata, Fukui, Osaka, and Nagasaki registries additionally confirm the vital status of patients using linkage to the residential database. We excluded data that were registered using death certificate only cases from the analysis.

Grouping of morphology was defined according to Cancer Incidence in Five Continents, Volume IX.^21^ Morphology codes of 8041–8045 and 8246 were defined as SCLC, and all SCLC patients (*n* = 10,911) were included in the study. Lung cancer patients with other morphologies were excluded. Disease stage at diagnosis was categorized using a summary staging system.^22^ LD-SCLC and ED-SCLC were defined using the Veterans Administration Lung Cancer Study Group (VALSG) staging system.^9^ In short, SCLC confined to one hemithorax was defined as LD. Ipsilateral lymph node metastasis and contralateral hilar lymph node metastasis was defined as ED. LD-SCLC was defined as localized and regional stage on the summary staging system. ED-SCLC was defined as distant stage on the summary staging system. Localized and regional stages correspond to T1-2, N0-2, and M0 in the American Joint Committee of Cancer (AJCC) TNM staging system.

This study was approved by the ethics committee of Osaka Medical Center for Cancer and Cardiovascular Diseases (Osaka, Japan) in September 2013. Use of the data was approved by the six prefectural cancer registries.

### Statistical analysis

We defined three periods (period 1, 1993–1998; period 2, 1999–2001; period 3, 2002–2006) to mirror the development of SCLC treatment. Elderly lung cancer patients were often defined as those who were aged 65, 70, or 75 years and older in clinical research. Therefore, age at diagnosis was classified into three groups: less than 65 years, between 65 and 74 years, and 75 years or older.

**Figure 1.**
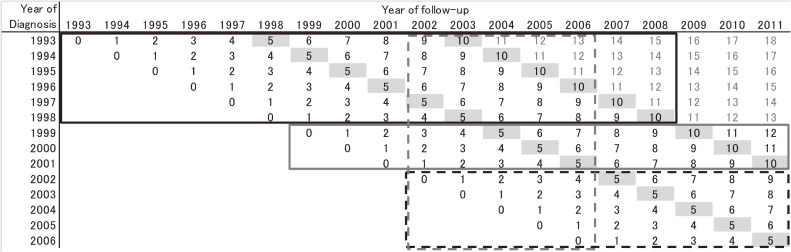
Patient data used in the survival analysis. Black figures indicate the data from six prefectural cancer registries, and the numbers within the cells indicate years of follow-up. Data in the black and gray solid frames were used to calculate 10-year relative survival by the cohort approach for patients diagnosed in period 1 (1993–1998) and period 2 (1999–2001), respectively. Data in the black dashed frame were used to calculate 5-year relative survival by the cohort approach for patients diagnosed in period 3 (2002–2006). Data in the gray dashed frame were used to calculate 10-year relative survival using period analysis for patients diagnosed in period 3 (2002–2006).

Trends in SCLC survival were assessed using relative survival (RS), because this is a standard method used to adjust for competing causes of death.^23^ RS is the ratio of the observed (overall) survival and expected survival. The background mortality of cancer patients was derived using the complete national population life tables by birth year, age, and sex.^24^ We estimated RS by applying the maximum likelihood method proposed by Esteve et al.^25^ We calculated the 1-, 3-, 5-, and 10-year RS for patients diagnosed in period 1 and period 2 using a conventional approach (cohort approach). Instead of 10-year RS, 1-, 3-, and 5-year RS for patients diagnosed in period 3 were calculated using the cohort approach, because 10-year survival data for these patients were not available (Figure 1, black dash frame). One-, 3-, 5-, and 10-year RS for patients diagnosed in period 3 were estimated using the period approach. Long-term RS could be estimated using the period approach from recently followed-up data. Ten-year RS for patients diagnosed in period 3 (2002–2006) was estimated using the survival data for patients diagnosed between 1993 and 2006 and followed-up between 2002 and 2006 (Figure 1, gray dashed frame).

RS was compared using the excess hazards model,^26^ a multivariate regression approach based on generalized linear models which adopts the Poisson assumption for the observed number of deaths. The excess hazards model is based on the idea that the total mortality hazard of cancer patients is decomposed into an *excess* hazard of death from cancer, and a hazard for other causes of death, derived from population life tables as background mortality of general populations. Period, sex, and age at diagnosis were included in the excess hazard model.

Using data of patients diagnosed in period 3, conditional 5-year survival was calculated. Conditional 5-year survival was 5-year survival with the pre-condition of having already survived a certain length of time (0 to 5 years in this report). Conditional 5-year survival for x-year survivors is calculated as follows: divide the (x+5)-year cumulative survival rate by the x-year cumulative survival, or calculate (x+5)-year cumulative survival, limited to the x-year survivors, in accordance with other studies.^27^^–^^30^

All analyses were conducted using STATA version 14.2 (StataCorp, College Station, TX, USA). The *strel* command in this software was used to calculate RS in both the cohort and period approaches.^31^

## RESULTS

In total, 98,475 lung cancer patients, including 10,911 SCLC patients, were registered in the six prefectural cancer registries between 1993 and 2006. Proportions of SCLC in periods 1, 2 and 3 were 11.6%, 11.1%, and 10.7%, respectively. Characteristics of SCLC patients in the three periods are shown in Table 1. Proportions of female patients were approximately 18% throughout the periods. Proportions of elderly patients (aged ≥75 years) in periods 1, 2, and 3 were 25.2%, 29.1%, and 33.3%, respectively. The proportion of patients with ED-SCLC increased from 42.9% in period 1 to 50.0% in period 3.

**Table 1.** Characteristics of study subjects

	1993–1998		1999–2001		2002–2006		Total	
	Number	%	Number	%	Number	%	Number	%
Sex								
Male	2,817	82.2	2,501	81.2	3,639	82.6	8,957	82.1
Female	610	17.8	579	18.8	765	17.4	1,954	17.9
Age, years								
≤64	1,093	31.9	842	27.3	1,169	26.5	3,104	28.4
65–74	1,469	42.9	1,341	43.5	1,770	40.2	4,580	42.0
≥75	865	25.2	897	29.1	1,465	33.3	3,227	29.6
Disease stage								
Limited disease (LD)	1,482	43.2	1,369	44.4	1,756	39.9	4,607	42.2
Extensive disease (ED)	1,469	42.9	1,371	44.5	2,203	50.0	5,043	46.2
Unknown	476	13.9	340	11.0	445	10.1	1,261	11.6
Total	3,427	100.0	3,080	100.0	4,404	100.0	10,911	100.0

**Figure 2.**
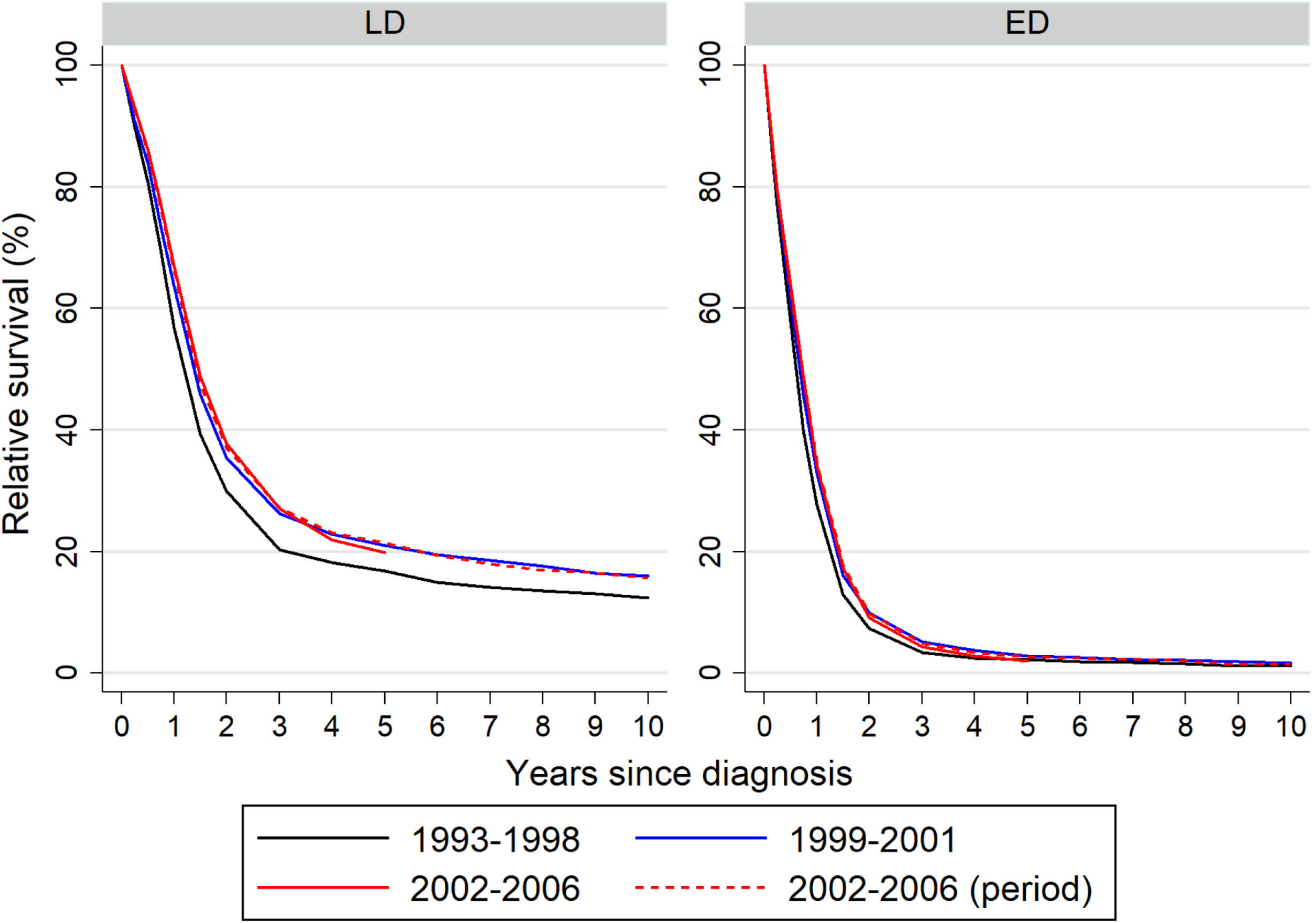
Ten year relative survival of patients with small-cell lung cancer. Relative survival was stratified by disease stage. ED, extensive disease; LD, limited disease.

Ten-year RS curves of each period by disease stage are shown in Figure 2 and Table 2. Five-year RS curves of both LD- and ED-SCLC patients in period 3 calculated using the cohort approach were similar to those estimated by the period approach. One-, 3-, 5-, and 10-year RSs of LD-SCLC patients in period 2 were better than those in period 1. The 10-year RS curve of LD-SCLC patients in period 3 estimated using the period approach was similar to that in period 2 estimated using the cohort approach. One- and 3-year RSs of ED-SCLC patients in period 3 were better than those in period 1, whereas 5- and 10-year RSs of ED-SCLC in period 1 were similar to those in period 3 estimated using the period approach. Ten-year RS curves in each period were similar between male and female patients with SCLC. RS of SCLC patients aged 75 years and more was approximately half that of patients aged less than 65 years in each period (eTable 1).

**Table 2.** 1-, 3-, 5-, and 10-year relative survival of patients with SCLC stratified by disease stage

Relative survival (%)	Years since diagnosis							
	1		3		5		10	
			
	Survival	(95% CI)	Survival	(95% CI)	Survival	(95% CI)	Survival	(95% CI)
**Limited Disease (LD)**								
Period 1 (1993–1998)	56.8	(54.3–59.1)	20.3	(18.3–22.3)	16.8	(14.9–18.7)	12.4	(10.6–14.4)
Period 2 (1999–2001)	63.6	(60.5–66.6)	26.2	(23.4–29.1)	21.1	(18.4–23.8)	16.1	(13.5–18.8)
Period 3 (2002–2006)	66.9	(64.5–69.2)	27.0	(24.8–29.3)	19.9	(17.8–22.0)		
Period 3 (period^a^)	66.2	(63.8–68.5)	27.2	(25.0–29.5)	21.4	(19.3–23.6)	15.6	(13.4–18.0)

**Extensive Disease (ED)**								
Period 1 (1993–1998)	27.7	(25.6–29.8)	3.4	(2.6–4.4)	2.3	(1.6–3.1)	1.2	(0.7–1.8)
Period 2 (1999–2001)	33.0	(30.1–35.9)	5.2	(3.9–6.7)	2.8	(1.9–4.0)	1.7	(1.0–2.9)
Period 3 (2002–2006)	34.3	(32.3–36.4)	4.3	(3.5–5.3)	2.0	(1.4–2.7)		
Period 3 (period^a^)	34.8	(32.8–36.9)	5.0	(4.0–6.0)	2.7	(2.0–3.6)	1.4	(0.8–2.3)

CI, confidence interval; ED, extensive disease; LD, limited disease; SCLC, small-cell lung cancer.

^a^Relative survival and CIs were estimated using the period method. Survival data of patients followed between 2002 and 2006 were used.

We estimated the EHR of SCLC patients’ hazard of death from cancer within 5 years (Table 3). When stratified by disease stage, LD- and ED-SCLC showed similar trends. Excess mortality in periods 2 and 3 was significantly lower than that in period 1. Female LD-SCLC patients showed no statistically significant difference in mortality from male patients, whereas female ED-SCLC patients showed significantly better survival than male patients.

**Table 3.** Excess hazard ratio (EHR) of death by excess mortality model stratified by disease stage

	Limited disease (LD)			Extensive disease (ED)		
	
	EHR	95% CI	*P* value	EHR	95% CI	*P* value
Period						
1993–1998	1	Reference		1	Reference	
1999–2001	0.84	0.77–0.92	<0.001	0.86	0.80–0.94	<0.001
2002–2006	0.77	0.72–0.84	<0.001	0.85	0.79–0.90	<0.001
Sex						
Male	1	Reference		1	Reference	
Female	1.04	0.95–1.13	0.401	0.92	0.85–0.99	0.028
Age at diagnosis, years						
≤64	1	Reference		1	Reference	
65–74	1.29	1.19–1.40	<0.001	1.23	1.15–1.31	<0.001
≥75	1.92	1.76–2.10	<0.001	1.71	1.58–1.85	<0.001

CI, confidence interval; EHR, excess hazard ratio.

Period, sex and age were included in the model.

**Figure 3.**
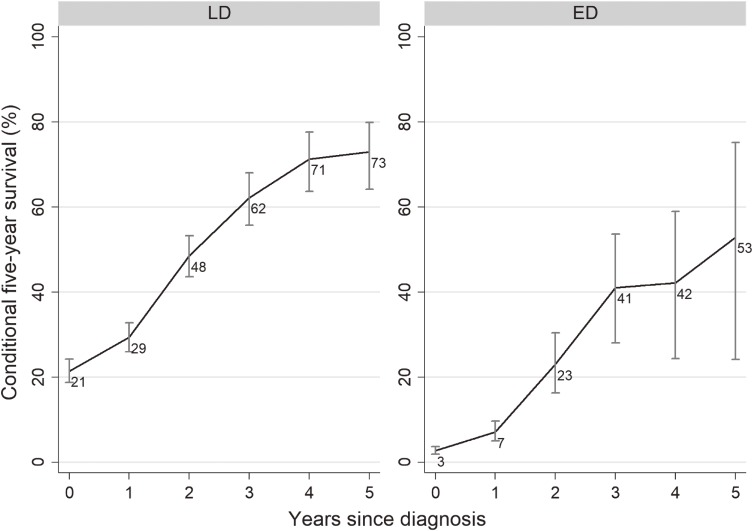
Conditional 5-year relative survival and 95% confidence intervals of SCLC patients stratified by disease stage. ED, extensive disease; LD, limited disease; SCLC, small-cell lung cancer.

Conditional 5-year RS stratified by disease stage are shown in Figure 3. Conditional 5-year survival for patients with LD-SCLC increased from 21% at year 0 to 73% at year 5, while that in patients with ED-SCLC increased from 3% at year 0 to 53% at year 5. Because the 2-year RS of patients with ED-SCLC was 9.8%, confidence intervals for the conditional 5-year survival of ED-SCLC patients at years 3 to 5 are wide.

## DISCUSSION

In this study, we found that the RS of patients with SCLC slightly improved between 1993 and 2006, despite increases in the number of elderly patients and relative proportion of ED-SCLC. This improvement in RS was confirmed after adjustment for period, sex, and age at diagnosis. To the best of our knowledge, this is the first study to show the RS of patients with SCLC stratified by disease stage using population-based data.

Among results, we found that the RS of patients with LD-SCLC in periods 2 and 3 were better than that in period 1. This improvement in survival was consistent with the development of chemoradiotherapy.^12^ A new chemoradiotherapy method, concurrent radiotherapy and hyperfractionated radiotherapy, improved the RS of patients with LD-SCLC diagnosed after 1997. LD-SCLC patients had a similar survival in period 3 to that in period 2. This seems consistent with the fact that no significant new treatment for LD-SCLC was developed during this time.

The improvement in ED-SCLC survival in periods 2 and 3 compared with period 1 was inconsistent with the development of chemotherapy. A clinical study in Japan showed that patients with ED-SCLC treated with the new combination of cisplatin and irinotecan had longer survival than those treated using standard chemotherapy with cisplatin and etoposide.^14^ In a replication study, however, cisplatin and irinotecan showed no significant benefit compared with standard chemotherapy.^32^^–^^34^ In addition, the higher rate of nonhematologic toxicity with the cisplatin and irinotecan regimen might decrease feasibility, and the new regimen might, therefore, have lacked impact on survival using population-based data. The RS of ED-SCLC patients in period 2 was better than that in period 1, despite no obvious improvement in ED-SCLC treatment. One reason might be the development of supportive care and palliative care. Total usage of opioids, a proxy for supportive care,^35^ was 706 kg of morphine equivalent in Japan in 1995, rapidly increasing to 891 kg in 2000 and 2,696 kg in 2004.^36^ Given that supportive care impacts the prognosis of patients with lung cancer, this increase in supportive care might have improved the prognosis of patients.^37^

The RS curves in period 2 were better than those in period 1 for both LD-SCLC and ED-SCLC. The RS curves in period 2 were similar to those in period 3 for both LD-SCLC and ED-SCLC. This similarity should be carefully considered because the improvement might have been due to stage migration. Improvements in diagnostic methods allow the detection of very small metastatic tumors. Patients with small distant metastasis would have been classified as LD in period 1. With the detection of small metastases with improved imaging, however, the patient would be diagnosed as ED. Movement of such patients with small metastases from LD to ED would improve the prognosis of LD patients, because their prognosis would be poorer than that of those without metastasis. Similarly, the prognosis of ED patients would be improved via the addition of patients with small metastases. The increased proportion of ED patients may support this hypothesis.

Conditional 5-year survival shows the conditional probability of surviving a further 5 years for cancer survivors.^38^ It is a more informative way for survivors to see their evolving prognosis over time. Conditional 5-year survival was low in our patients with LD-SCLC compared with other malignancies.^18^ Even 5 years post-diagnosis, conditional 5-year survival was 73%. The low conditional 5-year survival was mainly due to the poor prognosis of SCLC. In addition, the low conditional survival might be partly explained by the high proportion of heavy smokers among patients with SCLC.^39^ Even SCLC patients with long survival may eventually die due to other cigarette-associated disease and comorbidities.

Lung cancer screening might be another potential factor to influence SCLC survival. Because of aggressive growth of SCLC, most SCLC cases were discovered as symptomatic cancers during the interval of annual lung cancer screening,^40^ which suggests that lung cancer screening is unlikely to improve survival in patients with SCLC.^41^ Even if SCLC could be screened effectively, it is less likely that screening affects stage-specific survival. Therefore, lung cancer screening programs were unlikely to affect the results of our study.

The strength of this study is its use of population-based cancer registry data. Because all SCLC incident cases in six prefectures were included, the study is unlikely to have suffered from the selection bias which confounds clinical trials and hospital-based cancer registries. A second strength was its large sample size. Most reports of SCLC survival have been derived from hospital-based studies.^5^^,^^42^ The largest Japanese hospital-based lung cancer registry, the Japanese Joint Committee for Lung Cancer Registration, reported histology in specific lung cancer survival.^5^ However, their study included only 243 SCLC cases versus 10,911 incident SCLC cases in our present study.

This study has a number of limitations. First, long-term survival was estimated using data from only six prefectural cancer registries. Second, data quality was not particularly high. The proportion of death certificate only cases among registries was 3.1% to 24.6%. The generalizability of the results should, therefore, be interpreted cautiously. Thanks to the enactment of the Cancer Registry Law in 2013, the quality of population-based cancer registry data will shortly improve.^43^^,^^44^ This will allow new estimations of cancer survival with greater timeliness, longer follow-up, and inclusion of many more prefectures in Japan. Considering the decreasing trend in the incidence of SCLC,^45^^,^^46^ analysis might require larger coverage to attain a stable estimation. Third, detailed information, such as treatment, comorbidity, and smoking status, was not available. These variables affect cancer survival, but the data are not fully collected in population-based cancer registries. Verification of the influence of these clinical factors on prognosis would require studies using detailed clinical data from hospital-based cancer registries.

In conclusion, we reported the 10-year RS and conditional survival of patients with LD- and ED-SCLC. RS after 1999 was better than that before 1998, although conditional survival was poor even among the patients with LD-SCLC. The forthcoming improvement in the quality and timeliness of cancer registry data in Japan will allow continuous survey using population-based data from many prefectures to estimate the progress of treatment.

## APPENDIX A. SUPPLEMENTARY DATA

eTable 1. Relative survival of patients with SCLC stratified by sex and age
