# Supplementary material for: Trends in Small-Cell Lung Cancer Survival in 1993–2006 Based on Population-Based Cancer Registry Data in Japan
Source: J Epidemiol. 2019 Sep 5;29(9):347–53. doi: 10.2188/jea.JE20180112 (PMC6680055; doi:10.2188/jea.JE20180112)
Supplement: Supplementary file 1 [file je-29-347-s001.pdf]

eTable 1. Relative survival of patients with SCLC stratified by sex and age

Relative survival (%)	Years since diagnosis							
	1		3		5		10	
	Survival	95% CI	Survival	95% CI	Survival	95% CI	Survival	95% CI
Sex								
Male								
Period 1 (1993–1998)	41.4	(39.6 –43.1)	10.8	(9.7 –11.9)	8.6	(7.6 –9.6)	6.3	(5.3 –7.3)
Period 2 (1999–2001)	47.9	(45.6 –50.2)	15.0	(13.3 –16.7)	11.4	(9.9 –13.0)	8.6	(7.2 –10.2)
Period 3 (2002–2006)	47.7	(46.0 –49.4)	13.7	(12.5 –14.9)	9.6	(8.6 –10.7)		
Period 3 (period*)	48.0	(46.2 –49.6)	14.4	(13.2 –15.7)	10.9	(9.8 –12.1)	7.8	(6.7 –9.1)
Female								
Period 1 (1993–1998)	41.4	(37.7 –44.9)	12.9	(10.5 –15.5)	10.4	(8.2 –12.8)	7.6	(5.6 –9.9)
Period 2 (1999–2001)	46.2	(41.4 –50.9)	16.7	(13.3 –20.5)	12.0	(9.0 –15.4)	8.9	(6.2 –12.1)
Period 3 (2002–2006)	48.0	(44.3 –51.6)	15.3	(12.8 –18.0)	9.4	(7.4 –11.7)		
Period 3 (period*)	48.4	(44.7 –52.0)	16.5	(13.9 –19.3)	11.4	(9.1 –13.9)	8.1	(6.0 –10.5)
Age at diagnosis, years								
≤64								
Period 1 (1993–1998)	52.2	(49.5 –54.9)	14.0	(12.2 –16.0)	10.6	(9.0 –12.4)	8.4	(6.9 –10.2)
Period 2 (1999–2001)	59.5	(55.5 –63.3)	21.0	(17.9 –24.3)	17.3	(14.4 –20.5)	12.9	(10.3 –15.9)
Period 3 (2002–2006)	60.3	(57.4 –63.1)	18.6	(16.4 –20.9)	13.7	(11.7 –15.8)		
Period 3 (period*)	60.5	(57.5 –63.3)	19.7	(17.4 –22.1)	15.5	(13.4 –17.8)	11.7	(9.7 –14.0)
65–74								
Period 1 (1993–1998)	42.3	(39.9 –44.6)	11.1	(9.6 –12.7)	9.4	(8.0 –10.9)	6.1	(4.8 –7.5)
Period 2 (1999–2001)	48.4	(45.3 –51.4)	15.6	(13.4 –18.0)	10.6	(8.7 –12.7)	8.1	(6.3 –10.3)
Period 3 (2002–2006)	48.8	(46.4 –51.2)	13.3	(11.7 –15.0)	8.4	(7.1 –9.8)		
Period 3 (period*)	49.1	(46.7 –51.5)	14.6	(12.9 –16.3)	9.8	(8.4 –11.4)	6.5	(5.1 –8.2)
≥75								
Period 1 (1993–1998)	25.3	(22.6 –28.1)	7.7	(6.0 –9.6)	6.2	(4.6 –8.2)	3.9	(2.3 –6.2)
Period 2 (1999–2001)	34.2	(30.5 –37.9)	9.1	(6.8 –11.6)	6.8	(4.8 –9.2)	4.5	(2.5 –7.3)
Period 3 (2002–2006)	35.4	(32.8 –38.0)	10.8	(9.1 –12.7)	7.3	(5.8 –9.0)		
Period 3 (period*)	35.6	(32.9 –38.2)	10.9	(9.1 –12.9)	8.7	(6.9 –10.7)	5.0	(3.0 –7.8)

CI, confidence interval; SCLC, small-cell lung cancer.

* Relative survival and CIs were estimated using the period method. Survival data of patients followed between 2002 and 2006 were used.
